# Earthquake statistics changed by typhoon-driven erosion

**DOI:** 10.1038/s41598-020-67865-y

**Published:** 2020-07-02

**Authors:** Philippe Steer, Louise Jeandet, Nadaya Cubas, Odin Marc, Patrick Meunier, Martine Simoes, Rodolphe Cattin, J. Bruce H. Shyu, Maxime Mouyen, Wen-Tzong Liang, Thomas Theunissen, Shou-Hao Chiang, Niels Hovius

**Affiliations:** 1 Univ Rennes, CNRS, Géosciences Rennes - UMR 6118, 35000 Rennes, France; 2 Institut Des Sciences de La Terre Paris, ISTeP UMR 7193, Sorbonne Université, CNRS-INSU, 75005 Paris, France; 3 Helmholtz Centre Potsdam, German Research Center for Geosciences (GFZ), 14473 Potsdam, Germany; 4 Institute of Earth and Environmental Sciences, University of Potsdam, 14476 Potsdam-Golm, Germany; 5 Géosciences Environnement Toulouse (GET), UMR 5563, CNRS/IRD/UPS, Observatoire Midi-Pyrénées (OMP), 14 Avenue Edouard Belin, 31400 Toulouse, France; 6 Laboratoire de Géologie, École Normale Supérieure de Paris, 75231 Paris CEDEX 5, France; 7 Institut de Physique du Globe de Paris, Université de Paris, CNRS, 75005 Paris, France; 8 Géosciences Montpellier, Université Montpellier and CNRS UMR5243, 34090 Montpellier, France; 9 Department of Geosciences, National Taiwan University, Taipei, Taiwan; 10 Department of Space, Earth and Environment, Chalmers University of Technology, Onsala Space Observatory, Onsala, Sweden; 11 Institute of Earth Sciences, Academia Sinica, Taipei, Taiwan; 12 Department of Earth Science, University of Bergen, 5007 Bergen, Norway; 13 Center for Space and Remote Sensing Research, National Central University, Taoyuan City, 32001 Taiwan

**Keywords:** Natural hazards, Solid Earth sciences, Geodynamics, Geomorphology, Geophysics, Seismology, Tectonics

## Abstract

Tectonics and climate-driven surface processes govern the evolution of Earth’s surface topography. Topographic change in turn influences lithospheric deformation, but the elementary scale at which this feedback can be effective is unclear. Here we show that it operates in a single weather-driven erosion event. In 2009, typhoon Morakot delivered ~ 3 m of precipitation in southern Taiwan, causing exceptional landsliding and erosion. This event was followed by a step increase in the shallow (< 15 km depth) earthquake frequency lasting at least 2.5 years. Also, the scaling of earthquake magnitude and frequency underwent a sudden increase in the area where mass wasting was most intense. These observations suggest that the progressive removal of landslide debris by rivers from southern Taiwan has acted to increase the crustal stress rate to the extent that earthquake activity was demonstrably affected. Our study offers the first evidence of the impact of a single weather-driven erosion event on tectonics.

## Introduction

It is well established that tectonics and climate influence the evolution of the Earth’s surface by modulating the rate and pattern of erosion and sedimentation^1,2^. In turn, theoretical predictions and numerical models suggest that changes of surface topography by surface processes can promote tectonic deformation over geological times^3–5^ (1–10 Myr), enhance fault slip over intermediate time scales^6–9^ (1 kyr–1 Myr), and induce sufficient static stress changes over a seismic cycle (1–1,000 year) to trigger earthquakes^10^. However, the influence of ongoing surface processes on tectonics has not been directly observed. Over seasonal timescales, surface (un)loading induced by rainfall or snow can modulate local to regional seismicity^11,12,13^. Here we ask if a single erosional event can have a discernable effect on seismogenic processes, which dominate deformation of the Earth’s upper crust. Both large-magnitude earthquakes and extreme rainfall events can trigger instantaneous and widespread landsliding, driving the export of millions of tons of sediment from mountain areas over periods of months to years^2,14^. At these timescales, geophysical methods allow monitoring of changes in earthquake activity associated with erosional perturbations. Compared to large-magnitude earthquakes, rainfall events do not directly trigger aftershock sequences (even if surface (un)loading can modulate seismicity), which could preclude the observation of a seismicity change induced by erosion. We therefore focus in the following on detecting seismicity changes associated with an erosional perturbation induced by a rainfall event.

## Results

### Landslides and erosion triggered by typhoon Morakot in Taiwan

**Figure 1:**
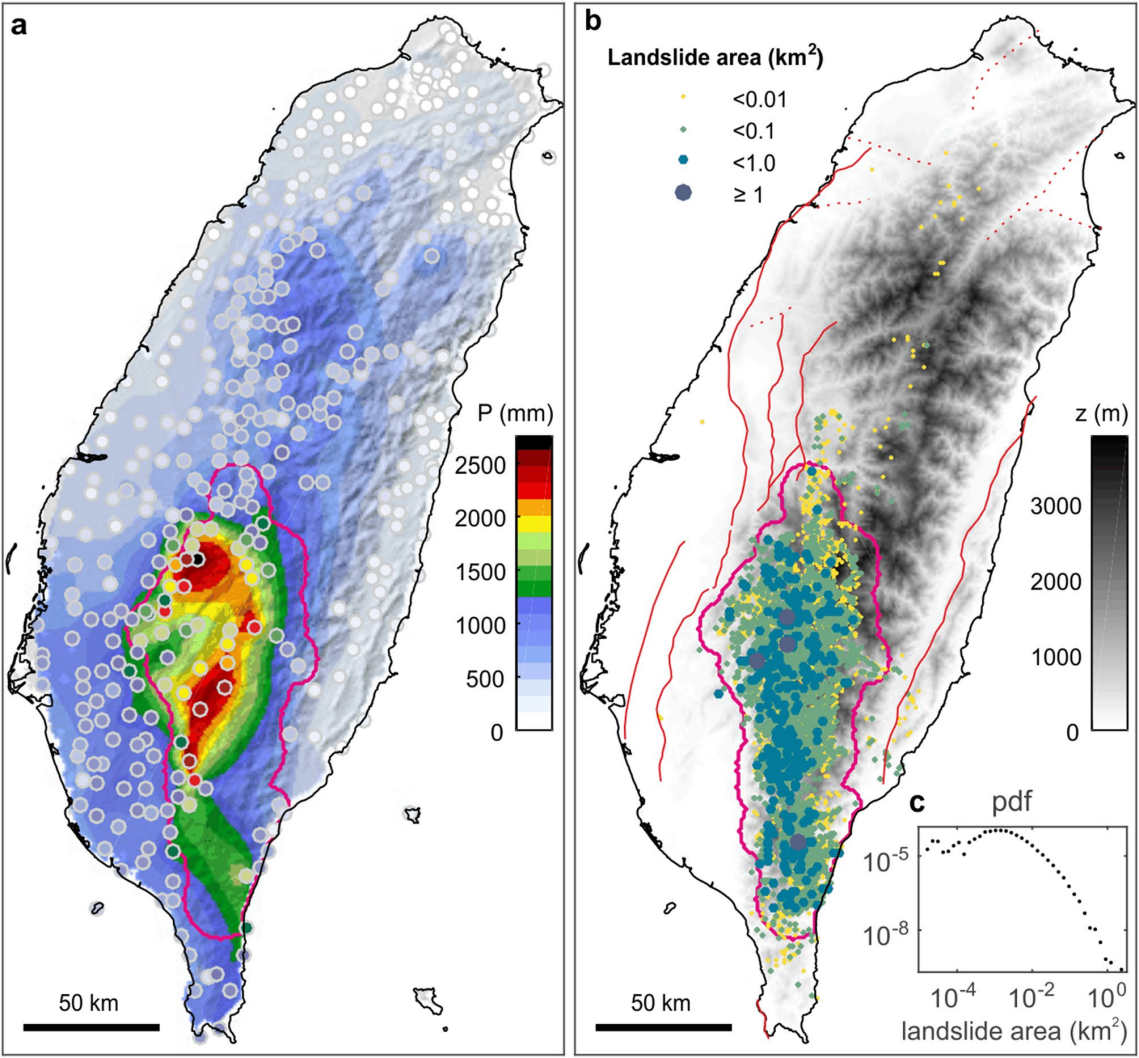
Morakot-driven rainfall and landslides in Taiwan. (**a**) Hillshaded map of cumulative rainfall during typhoon Morakot (7–9 August 2009), obtained by interpolation of data from local weather stations (colored dots). (**b**) Digital elevation model of Taiwan with location of mapped landslides triggered by typhoon Morakot. Circle size and color indicate the surface area of a landslide, while the magenta line delimits the area with highest spatial density of landslides (see “Methods” section, Supplementary Fig. S2), referred to as the landsliding zone. Solid and dashed red lines indicate active thrust and strike-slip or normal faults, respectively^22^. Other less well identified faults exist inside the range^27^. (**c**) Probability density distribution of the surface area of landslides triggered by typhoon Morakot for areas greater than 10 m^2^. Maps were performed using Matlab R2019b.

For an example of an erosional perturbation, we consider typhoon Morakot, which made landfall in Taiwan from 7 to 9 August 2009. It delivered up to 3 m of precipitation in 3 days (Fig. 1), the largest recorded rainfall event in Taiwan in the past 50 years^15^. The typhoon triggered more than 10.000 landslides (see “Methods” section) in mountainous southwest Taiwan, where cumulative rainfall exceeded ~ 1 m (Fig. 1, Supplementary Fig. S1). In this area of ~ 7,000 km^2^ (hereafter the landsliding zone), that accounts for ~ 99% of the 1.2 km^3^ of total landslide volume^16^, the landslide spatial density ranges between 4 and 22 km^-2^ (see “Methods” section, Supplementary Fig. S2). The equivalent average erosion induced by these landslides is ~ 17 cm, which corresponds to ~ 10–100 years of erosion at the decadal average rate^2^. Most triggered landslides were connected to the river network^17^, which has led to a sharp increase of suspended sediment export after Morakot^18,19^. Consistent with geomorphological observations after the 1999 Mw 7.6 Chi-Chi earthquake^20^, enhanced sediment removal persisted for > 2 years^18,19^, although export of the coarse fraction of landslide debris may take decades or more^21^. The exact volume of sediment export is difficult to estimate, but the landsliding zone must have undergone a progressive surface mass unloading after one of the largest weather-driven erosion events on record. Despite a very likely large uncertainty, we roughly estimate that about 20–50% of the 1.2 km^3^ of landslide volume was exported of the landsliding zone after typhoon Morakot over the time period 2009–2012 (see “Methods” section).

The landsliding zone belongs to a tectonically active region and is bounded in the east and west by several identified active thrust faults^22^. Thrust faults located in the western foothills have a dip angle between 10° and 30° and merge at depth, probably around ~ 10–15 km, into a basal decollement beneath the range^22,23^. In the east, the Longitudinal Valley fault has a dip angle ~ 45°–60°. Together, these faults accommodate ~ 40 mm year^−1^ of shortening, which is about half of the total convergence rate across the Taiwan plate boundary^24^. In addition, less well constrained faults are located beneath the range, as testified by the frequent seismicity observed in Central Taiwan.

### Temporal changes in seismicity after Morakot

To determine how the erosional unloading due to typhoon Morakot may have impacted fault dynamics, we analyze the evolution of shallow (< 15 km) seismicity in Taiwan after it made landfall. Because the expected stress change is small compared to background tectonic loading at seismogenic depth^10^, we focus on detecting changes in the statistics of recorded seismicity, such as the earthquake frequency, seismic moment rate and the b-value of the Gutenberg–Richter earthquake size distribution (see “Methods” section), rather than on individual events. We use the seismicity catalogue of the Central Weather Bureau of Taiwan, which includes > 340,000 earthquakes during the period 1995–2015 over Taiwan island.

**Figure 2:**
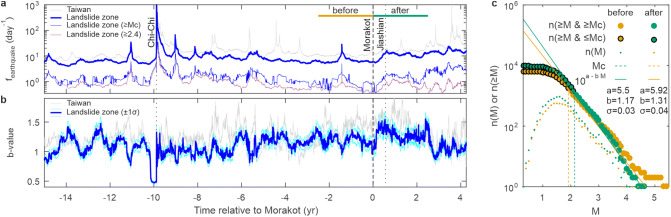
Time evolution of seismicity in Taiwan relative to typhoon Morakot. (**a**) Time evolution of frequency of shallow (< 15 km) earthquakes. The thick and thin blue line indicates the frequency of all earthquakes and of earthquakes greater than the completeness magnitude, respectively, inside the landsliding zone. The magenta line indicates the frequency of earthquakes inside the landsliding zone with a magnitude greater than a conservative value of 2.4 for the completeness magnitude. The thin grey line indicates the frequency of all earthquakes outside the landsliding zone. (**b**) Time evolution of the b-value of the Gutenberg-Richter law inside (heavy blue line) and outside (light grey line) the landsliding zone (see “Methods” section, Supplementary Fig. S1). (**c**) Gutenberg-Richter law fits over the distributions of cumulative earthquake numbers in the landsliding zone as a function of earthquake magnitude during the 2.5 years before (yellow) and after (green) typhoon Morakot (see “Methods” section).

First, we assess the time evolution of earthquake statistics using a temporal sliding window of 1,001 earthquakes (see “Methods” section). Results show a stepwise increase of the frequency of shallow (< 15 km) earthquakes in the landsliding zone after Morakot (Fig. 2, Supplementary Fig. S3). Although this frequency increase is orders of magnitude lower than after the Chi-Chi earthquake, it is observed both for earthquakes with magnitude above the completeness magnitude (from ~ 0.8 to ~ 2 earthquakes per day) and for all recorded earthquakes (from ~ 5 to ~ 10 earthquakes per day). The increase of earthquake frequency during the 2.5 year after Morakot has a probability of 1 (see “Methods” section, Supplementary Fig. S4). The probability is still significant, at the 95% confidence interval, when considering a frequency increase by a factor ~ 1.2–1.5. Except for the regional seismicity rate increases due to the Chi-Chi earthquake, this is the only significant increase in earthquake frequency, over a period of 2.5 year, observed over the investigated period (1994–2013).

**Figure 3:**
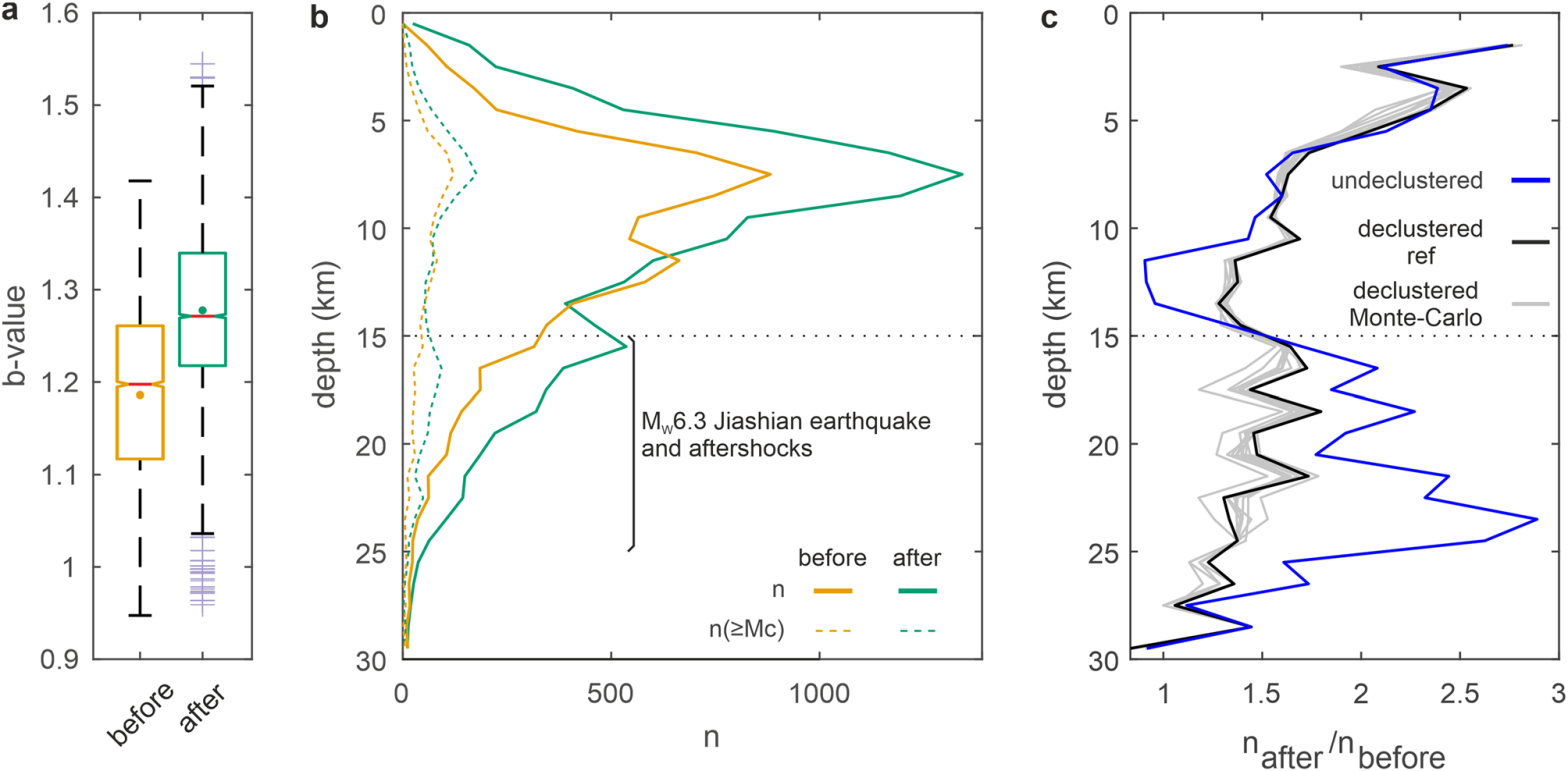
Change in b-value and depth of earthquakes after typhoon Morakot. (**a**) Notched whisker plots of b-value estimates for 2.5 years before (yellow) and after (green) typhoon Morakot inside the landsliding zone show the median (red line), mean (dot), 25th and 75th percentiles (box limits), whisker lengths (dashed lines) and outliers (purple crosses) of the b-value. Notches display the variability of the median between samples. (**b**) Histograms of earthquake depth during the 2.5 years before (yellow) and after (green) typhoon Morakot in the landsliding zone. Solid and dashed lines indicate depth-distribution for earthquakes of all magnitudes and magnitudes greater than the completeness magnitude, respectively. (**c**) Depth distribution of the ratio of the number of earthquakes in the 2.5 years after typhon Morakot, n_after_, over the number of earthquakes in the 2.5 years before, n_before_. The blue line indicates results considering all earthquakes, the black line indicates results considering the reference declustered catalog and the grey lines indicate results obtained using the 50 declustered catalogues, resulting from a Monte-Carlo sampling of the model parameter space (see “Methods” section).

Moreover, the increase in earthquake frequency after Morakot is associated with an increase in the b-value from 1.18 ± 0.1 to 1.28 ± 0.1 (Fig. 3a). Both increases have a step-like shape, which lasts for at least 2.5 years (Supplementary Figs. S5, S6) and do not follow an Omori-type inverse law, which describes the temporal evolution of aftershock sequences. Because the seismometer network used to detect earthquakes remained similar in the time period January 2007 to December 2011 (see “Methods” section, Supplementary Fig. S7), we restrict our comparison to the time period from 2.5 years before to 2.5 years after Morakot. More instruments were added, mainly in North Taiwan, at the beginning of 2012, which explain why the frequency of earthquakes remains high in the landsliding zone after 2012, despite a decrease of the b-value towards its pre-Morakot value. Using a conservative and constant value for the completeness magnitude of 2.4 (see Fig. 2, Supplementary Fig. S3), we observed that earthquake frequency decreases to pre-Morakot values after about 2.5. The increase of the b-value in the landsliding zone after Morakot is found for different fitting methods of the Gutenberg-Richter law and sampling methods associated with the sliding time window (see “Methods” section, Supplementary Figs. S5, S6) and therefore deemed robust.

In addition, considering all earthquakes during the 2.5 years before and after Morakot gives b-value estimates of 1.17 ± 0.03 and 1.31 ± 0.04 (Fig. 2c), respectively, similar to the values obtained by averaging the temporal b-value signal (Fig. 3a). We observe that the increase in earthquake frequency and b-value in the landsliding zone after Morakot coincides with an increase in the number of shallow earthquakes at depths < 15 km (Fig. 3b,c). Moreover, the increase is more pronounced closer to the surface and reaches a factor 2 to 3 in between 0 and 5 km of depth. We also observe a secondary peak at greater depth, 15 to 25 km. However, the rate of seismic moment release remains low after typhoon Morakot, and potentially lower than before (Supplementary Fig. S3). Crucially, earthquakes outside the landsliding zone do not show a significant temporal increase of both their frequency and b-value after Morakot.

### Seismicity changes are not associated to earthquake clustering

These temporal changes in earthquake statistics after Morakot are determined from an undeclustered earthquake catalog. However, using a declustered catalog (see “Methods” section) also leads to similar changes in earthquake frequency and in its associated probability, b-value and depth-distribution after Morakot (Fig. 3c, Supplementary Figs. S9, S10). Moreover, this result is not limited to a single parametrization of the declustering algorithm and appears robust for all the acceptable combinations of declustering parameters. For instance, the increase in the number of earthquakes after Morakot at 15 to 25 km of depths is fully removed when considering any of the resulting declustered catalog, while the shallow peak remains (Fig. 3c). This demonstrates that these seismicity changes at shallow depths are not associated with triggering processes by large mainshocks, or by other earthquakes. In addition, based on comparisons between the observed earthquake catalog and synthetic catalogs that share the same average properties, we demonstrate that the observed changes in earthquake frequency and b-value after Morakot, in the landsliding zone, depart statistically from random temporal changes (Supplementary Fig. S12).

### Spatial changes in seismicity after Morakot

**Figure 4:**
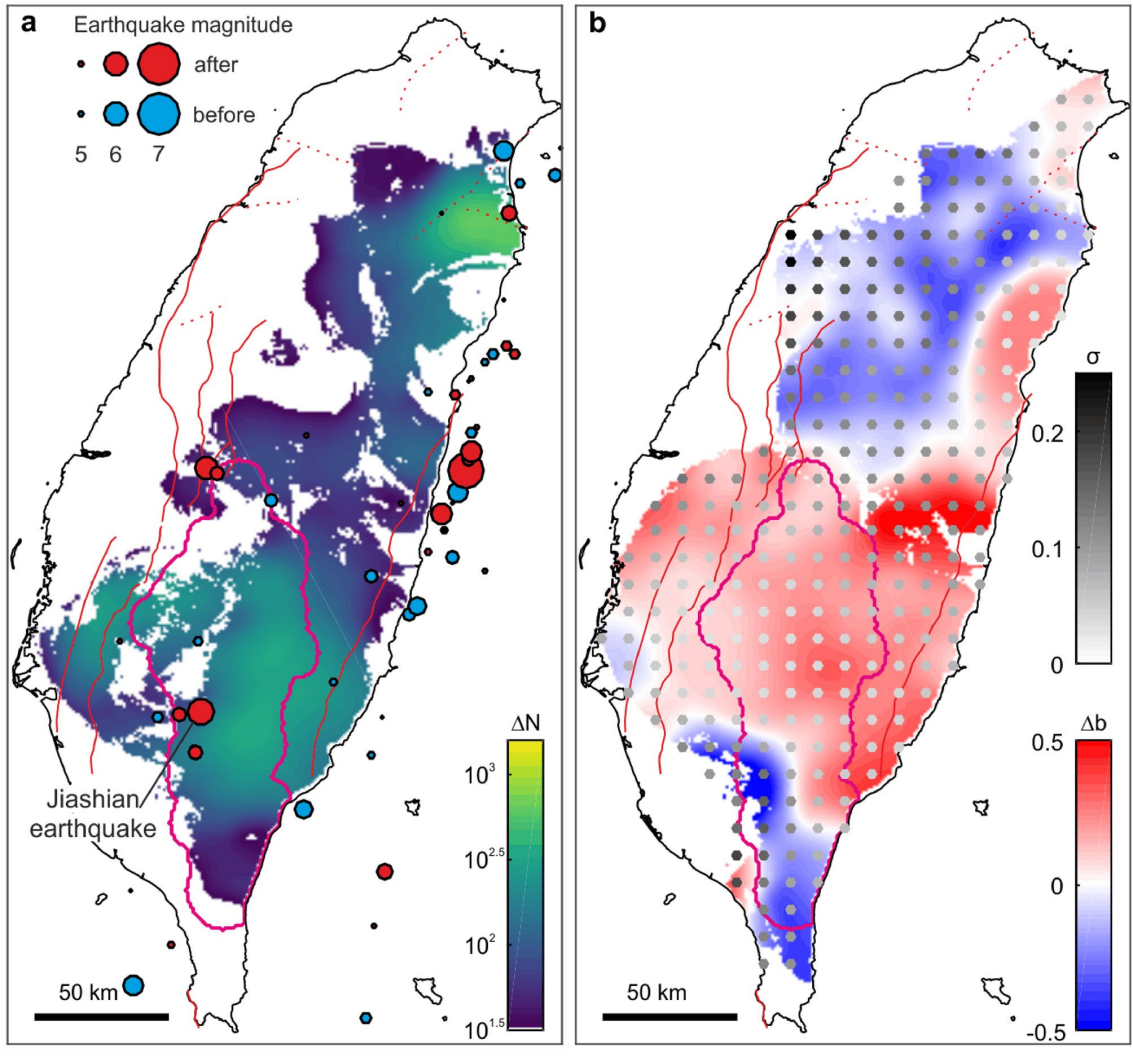
Changes in seismicity after typhoon Morakot. (**a**) Map of difference of shallow (< 15 km) earthquake numbers, $\Delta N$, during the 2.5 years after and before typhoon Morakot. Only earthquakes greater than the completeness magnitude were considered. For readability, $\Delta N$ values lower than 10^1.5^ are shown in white. Red and blue circles locate earthquakes greater than magnitude 5 after and before typhoon Morakot, respectively. (**b**) Change in b-value, $\Delta b$ (red-blue colormap), and uncertainty, *σ* (gray circles) of b-value estimates (see “Methods” section, Supplementary Fig. S3). Maps were performed using Matlab R2019b.

To assess how the chosen delimited area affects our results, we compute the change in the spatial pattern of the frequency and b-value of shallow (< 15 km) earthquakes from before to after Morakot. For this, we use a sliding window in space with a radius of 30 km, which allows us to detect large-scale features not affected by small sub-samples of events (see “Methods” section). It is applied separately to earthquakes in the 2.5 years before and after Morakot, respectively. Consistent with the temporal evolution of earthquake frequency, results show an increase in the number $N$ of earthquakes after the typhoon over the landsliding zone (Fig. 4, Supplementary Fig. S8). This increase is observed for earthquakes with magnitudes above the completeness magnitude and also for all recorded earthquakes. It is not limited to the vicinity of the landsliding zone, but it also occurred in northeast Taiwan. However, outside the landsliding zone, increases in the b-value appear not to be associated to increases in the number of earthquakes. For instance, North-East Taiwan displays a significant increase in the number of earthquakes associated to a decrease of the b-value. We note that the spatial correlation between earthquake statistics change and the landsliding zone is less well resolved and less robust (see “Methods” section) than the temporal correlation.

## Discussion

Previous intense erosional events associated with the Chi-Chi earthquake (1999), and typhoons Herb (1996) and Toraji (2001) did not induce any detectable change of seismicity. This may be because these events had less erosion, the total volume of ~ 0.45 km^3^ of landslides triggered by Chi-Chi earthquake^25^ being by far the largest, and because most landslides triggered by these events deposited debris distant from rivers^14^. Besides, the high rate of aftershocks after Chi-Chi prevents detection of a change in earthquake frequency or b-value associated with erosion due to Chi-Chi or Toraji, if there was any. We also note that the changes in earthquake frequency and b-value are not perfectly synchronous with typhoon Morakot and occur about 1–2 months after the typhoon. However, the time resolution of our temporal analysis does not allow us to detect with confidence changes occurring with a period less than about 100 days (see Supplementary Fig. S5b). The duration of the transient increase in earthquake frequency and b-value occurring after typhoon Morakot lasts about 2.5 years.

Non-erosional causes of the observed changes in earthquake statistics in SW Taiwan are possible but appear less likely. Earthquakes result from the stresses induced by tectonics, in general, but also by other earthquakes^26^. The 4th of March 2010, the Mw 6.3 Jiashian earthquake occurred within the landsliding zone, close to its western limit, and was followed by many aftershocks. However, this earthquake and most aftershocks are located at 15–25 km depth (Supplementary Fig. S11), as shown by seismological records^27^ and further confirmed by the declustering process, which mostly removes events below 15 km depth, (Fig. 3, Supplementary Fig. S10). Therefore, the Jiashian earthquake does not affect shallow earthquake statistics and cannot be considered responsible for the increase of the b-value after Morakot (Supplementary Fig. S10).

Hydrological triggering of seismicity after the heavy rainfall during typhoon Morakot, either by surface loading or by pore pressure diffusion^28^, could be an alternative mechanism. Indeed, on the east coast of Taiwan, where landslide erosion was limited, borehole water levels rose by 4 m after typhoon Morakot, and a volumetric contractional strain was observed^29,30^. However, both signals decayed in ~ 6 months and their amplitudes and temporal evolutions do not depart from the mean seasonal trends observed from 2006 to 2011. Moreover, pore pressure diffusion along permeable faults after large rainfall events generally leads to episodic increases of seismicity^31^ and not to prolonged changes as observed in our case.

The temporal and spatial collocation of intense landsliding triggered by typhoon Morakot and the observed increase of shallow earthquake frequency and b-value suggest a potential mechanistic link. Direct physical modeling of the impact of erosion during and after Morakot on seismicity is beyond our reach because the location, magnitude and rate of sediment export from the landsliding zone are not reliably constrained. Despite this, simple elastic models show that large erosional events with rapid sediment export can induce static stresses at depth, sufficient to modulate tectonic stresses on the shallower (< 5–10 km) parts of faults^10^. Removing 20 to 50% of the landslide volume in 2.5 years, equivalent to about 2 to 5 cm of average erosion over the landsliding zone, would lead to a Coulomb stress increment on a nearby thrust fault of about 0.1 10^–1^ to 0.25 10^–1^ bar at 5 km depth^10^, using a thrust dip angle of 30° and an effective friction of 0.6. These stress increments are roughly similar to the ones induced by seasonal hydrologic loading in the Himalaya, ~ 0.2–0.4 × 10^–1^ bar, which are suggested to lead to a seasonal modulation of earthquake frequency^32^.

In addition, spring-slider models^33^ and 2D elasto-dynamic models of seismogenic faults^34^ with rate-and-state friction laws^35,36^ show that the rate of seismicity can increase linearly or more than linearly due to a positive, step-like stress perturbation. It is also observed that shallow earthquakes generally have smaller magnitudes and larger b-values than deeper earthquakes^37^ possibly because they nucleate at lower differential stresses^38^. Our observations suggest that the intense and prolonged sediment export and surface unloading after typhoon Morakot could have acted as a progressive increase of stresses, with a complex spatio-temporal evolution, on the shallow parts of underlying thrust faults^10^, giving rise to a similar increase of shallow earthquake frequency and b-value in Southwest Taiwan.

The proposed erosional-driven mechanism to explain the changes in seismicity after Morakot offers new perspectives on the links between climate, erosion and tectonics^4,5^ at the time scale of elementary processes. While numerous studies have shown that earthquakes and storms can trigger landslides^2,14,20,25^, this is to our knowledge the first potential evidence of the short-term and ongoing influence of erosion on seismicity. Because the mechanical link between erosion and stresses is through crustal elasticity^10^, crustal deformation is sensitive not only to extreme weather-driven erosion but also to the cumulative effects of smaller but numerous erosion events. More frequent extreme rainfall, for instance under a warmer climate^39^, could result in accelerated sediment transport^19^ and in turn in changes in the rate of shallow earthquakes, similar to the modulation of seismicity by surface water load variations^13^. Over the duration of the transient increase in seismicity, the shallow but small-magnitude seismicity induced by erosion is not likely to trigger new landslides, and the seismic moment rate would not be significantly affected. Hence, we do not expect a significant feedback of this additional deformation on erosion. However, the impact of this short-term transient signal on the seismicity and erosion over longer time scales, such as the duration of a seismic cycle, remains to be investigated. Our results therefore call for a new generation of process-based models coupling landscape dynamics^21^ and fault dynamics^34^ at scales relevant to natural hazards and societal issues. In these models, storms, floods, mass wasting, river sediment transport, redistribution of water, elastic stress transfer, seismicity and seismic wave propagation should all be represented to account for the complexity of the links between climate, erosion and tectonics.

## Methods

### Morakot rainfall data

The map of cumulative rainfall during Morakot was obtained by natural-neighbor interpolation of cumulative hourly rain gauge measurements over the period 7 to 9th of August 2009. Data from 377 stations across Taiwan were used from the Data Bank for Atmospheric Research at the Taiwan Typhoon and Floods Research Institute.

### Morakot landslide catalogue

Mapped landslides were delineated manually by comparing surface reflectivity and morphology on pre- and post-event FORMOSAT-2 satellite images^40^ (2 m panchromatic and 8 m multi-spectral). To cover most of the islands we mosaicked multiple cloud-free pre-event (01/14, 05/08, 05/09, 05/10, 06/06) and post-event (08/17, 08/19, 08/21, 08/28, 08/30, 09/06) images taken in 2009. For parts of the inventory, especially east of the main divide, landslides were first mapped automatically and then edited manually. For both approaches, the scar, runout and deposit areas are not differentiated. We did not consider debris flow transport areas and excluded gentle slopes (< 20°) from mapping to avoid confusion with human activity. Special attention was given to the separation of individual landslides, which had common transport or deposit areas but independent initiation points^41^. The robust and conservative estimation of landslide surface area is especially important for the estimation of landslide volume^42^.

### Estimation of landslide volume

The landslide volume was estimated based on landslide area, following the method of ref. 16 and 41. Briefly, we assumed constant size ratios between scar and deposit areas of 1.1 and 1.9 for mixed and bedrock landslides, respectively^42^. Then, we converted the scar area into volume using a power law with different prefactor ($\alpha$) and exponent ($\gamma$) for mixed and bedrock landslides, with $\alpha =0.146$ and $\gamma =1.332$ for ${A}_{scar}<1e5$ m^2^ and $\alpha =0.234$ and $\gamma =1.41$ for ${A}_{scar}>1e5$ m^2^, respectively^41^. Recent studies have proposed a regional scaling relationship for southern Taiwan, based on measurement of large landslides caused by typhoon Morakot^43^. With these parameters ($\alpha =0.202$, $\gamma =1.268$), and without scar correction, we obtain volumes ~ 3 times larger for intermediate size (mixed) landslides and twice smaller volume for very large (bedrock) landslides. Overall, this would not change the order of magnitude of erosion nor the results discussed in this study. Distributing uniformly the total volume, ~ 1.2 km^3^, of landslides triggered by typhoon Morakot over the area of the landsliding zone, ~ 7,000 km^2^ , gives about 17 cm of thickness of mobilized sediments.

### Estimation of sediment export after typhoon Morakot

There is currently no method allowing a robust and accurate measurement of sediment transport after typhoon Morakot, including suspended and bed loads, at the scale of Taiwan. Concerning suspended load, about 0.75 GT of sediments have been exported away in 2009 from the Gaoping catchment compared to an average ~ 0.02 GT/year over the period 1950–2008 (ref.^19^). Most of the 0.75 GT of sediment export in 2009 from the Gaoping catchment, which drains a large area of the west part of the landsliding zone, can therefore be attributed to the impact of the landslides triggered by Morakot. Similarly, over the Island of Taiwan, suspended sediment discharge increased from about a mean of ~ 0.38 GT/year to ~ 1.1 GT/year in 2009, and a large part of this increase can be attributed to the impact of Morakot^19^. In 2010, suspended sediment discharge decreases to pre-Morakot values, partly due to the absence of large water discharge events. Timescales for bedload sediment export is probably much larger, between a few years to decades^21^ and potentially centuries. Most rivers connected to the landsliding zone experienced rapid and intense sediment aggradation, with 10–100 m of sediment thickness, in response to Morakot typhoon. To our knowledge, no study has been published on the regional evolution of this bedload sediment mass. The estimated total volume 1.2 km^3^ of landslides triggered by Morakot could translate in about 2.5–3 GT of sediments. It is therefore likely that about 20–30% of this mass was removed by suspended transport in 2009, and probably a few percent or more in the following years. Therefore, a rough estimate could lead to possibly 20–40% of sediment export over the time period 2009–2012. Adding bedload transport could hypothetically increases this estimate to 20–50% for the time period 2009–2012.

### Earthquake catalogue of Taiwan

We extracted earthquakes at shallow depths (< 15 km) from the earthquake catalogue of the Taiwan Central Weather Bureau^44^ for the period 1995–2015 over the emergent part of Taiwan. This catalogue is accessible through the Taiwan Central Weather Bureau (https://gdms.cwb.gov.tw). The monitoring network includes short-period and broadband seismographic systems stations. The location and number of seismic stations changed during the period 1995–2015. The configuration of the seismic network in the south of Taiwan and in the landsliding zone remained relatively similar over the period January 2007 to December 2012 (see Supplementary Fig. S8), but after 2012, 2.5 years after Morakot, the number of stations was increased mostly in North Taiwan. This has caused a decrease of the measured completeness magnitude over all of Taiwan, including in the landsliding zone (see Supplementary Fig. S2). We therefore do not focus our analysis on changes in the frequency of earthquakes occurring after 2.5 years after Morakot (beginning of 2012). Yet, we note that using a constant and conservative value for the completeness magnitude of 2.4 (see Fig. 2, Supplementary Fig. S3), we observed that earthquake frequency decreases to pre-Morakot values after about 2.5 years.

### Characterization of earthquake size distribution

The Gutenberg–Richter distribution is classically used to characterize the relation of the number of earthquakes above a given magnitude, $n\left(\ge M\right)={10}^{a-bM}$, to the magnitude $M$, where a and b are parameters related to the number of earthquakes and to the slope of the relationship, respectively. This relationship is appropriate only for magnitudes above the completeness magnitude, ${M}_{c}$, of the catalogue. ${M}_{c}$ is determined by a modified version of the simple but robust maximum curvature method^45^, where ${M}_{c}$ is equal to the maximum of the first derivative of the frequency-magnitude curve, plus 0.5. We compute a maximum likelihood estimate^46^ of the b-value, $b={log}_{10}e/(\overline{M}-{M}_{c})$ and of its uncertainty, $\sigma =b/\surd n(\ge {M}_{c})$, where $\overline{M}$ is the mean magnitude of the considered earthquakes with $M\ge {M}_{c}$. Note that the number of earthquakes considered has a strong control on the b-value estimate and its uncertainty^47^, and that only relative spatial or temporal changes of the b-value should be interpreted.

### Time evolution of earthquake statistical properties

We use a temporal sliding window to subsample the earthquake catalogue and to assess time variations of earthquake statistical properties. Because the uncertainty of the b-value strongly depends on the number of considered events, and because larger samples give better estimates, we use a sliding window of $N=$ 1,001 events to prevent undue statistical bias. The window is centred on a given earthquake and the corresponding b-value and earthquake frequency are determined for the 500 earthquakes that occurred immediately before and immediately after this event. Earthquake frequency is computed by dividing $N$ by the temporal length of the sliding window. Among the 1,001 earthquakes, only those with magnitude above ${M}_{c}$ are used to estimate the b-value. We assess the effect of changing the sampling method of the temporal window, by considering an a priori value of ${M}_{c}=2.25$, and considering a fixed number of earthquakes with a magnitude ${>M}_{c}$ ranging from $N=$ 101 to $N=$ 1,001 (Supplementary Figs. S5, S6). We find a consistent increase of b-value in the 2.5 years after Morakot for $N$ ranging from 101 to 501, as all these earthquakes occurred within a time window duration lower than 2.5 years. For $N=751$ or $N=$ 1,001 the window duration is equal to or greater than 2.5 years (about 3 years for $N=$ 1,001), which leads to over-smoothing of the signal, and prevents detection of changes occurring on shorter time-scales. It is also notable that changing the fitting method from maximum likelihood to least-square, which is generally considered less reliable, does not significantly change the relative variation of the b-value in time (Supplementary Fig. S5). This includes the changes occurring after Morakot.

### Probability of earthquake frequency change with time

Following ref.^48^, we compute the probability *P* that the earthquake frequency increases by a factor greater than *r* between a period 1 and 2,

$$ P\left( {\frac{{\lambda _{2} }}{{\lambda _{1} }} > r} \right) = 1 - \frac{1}{{N_{2} !N_{1} !}}\int_{0}^{\infty } {e^{{ - x}} \Gamma } \left( {N_{2}  + 1,rx\frac{{\Delta t_{2} }}{{\Delta t_{1} }}} \right)x^{{N_{1} }} dx $$

where $\Gamma \left(n,x\right)={\int }_{0}^{\infty }{e}^{-t} {t}^{n-1} dt$ is the incomplete Gamma function, $N$ is the number of earthquake over a certain period $\Delta t$ and $\lambda =N/\Delta t$ is the earthquake frequency. The subscripts 1 and 2 refer to the time periods 1 and 2. We first apply this approach to determine the probability of earthquake frequency change between the 2.5 year before and after Morakot, in the landsliding zone considering only earthquakes with a depth shallower than 15 km (Supplementary Fig. S4a). We find a probability 1 for an increase of earthquake frequency (i.e. with $r>1$), when considering all the earthquakes or only earthquakes above the completeness magnitude. The 90% confidence interval, $0.05<P<0.95$, of an earthquake frequency change is found for $1.23<r<1.40$, when considering only the earthquakes above the completeness magnitude, and $1.45<r<1.52$ for all magnitudes. This demonstrates that the change of earthquake frequency after Morakot is significant with a ratio of 1.23, at least. We then apply the same analysis to the entire catalog by using a double sliding window of period 2.5 years after and before the center time (Supplementary Fig. S4b). The largest increase in earthquake frequency, with a factor close to 5 for a probability inside the confidence interval, is Chi-chi earthquake. Except for Chi-Chi, the time period including typhoon Morakot and Jiashian earthquakeis the only one associated with a significant and positive change of earthquake frequency over a period of 2.5 years since 1994. However, as demonstrated in this manuscript, Jiashian earthquake did not lead to a significant increase in the number of earthquakes at shallow depths (Fig. 3; Supplementary Figs. S9, S10, S11, S13).

### Spatial variations of earthquake statistical properties

We use a common spatial sliding window to subsample the earthquake catalogue and to assess variations of earthquake statistical properties in space^49^ between the 2.5 years before and after typhoon Morakot. We use a radius of 30 km for the sliding window, which enables sampling of a sufficiently large number of earthquakes at a length scale that is smaller than that of the landslide zone of 7,000 km^2^. The sampling window effectively corresponds to a disk shape extending to a depth of 15 km. The b-value is determined for the recorded earthquakes in each disk volume by maximum likelihood estimation. This method has an inherent statistical bias as the number of sampled earthquakes changes significantly depending on the local rate of seismicity. The minimum number of events for the determination of the b-value is set arbitrarily at 50. Our method can give rise to small-scale shapes in the maps of b-value, such as disk and rod shapes, that are not the focus of this study. We use a disk shape kernel with a radius of 15 km, convolved with the initial b-value map to blur the mapped patterns and to isolate features with longer wavelengths. More sophisticated methods exist to compute spatial variations of b-value, including a penalized likelihood-based method^50^ and a distance-dependent sampling algorithm^51^.

### Declustering and seismicity changes after Morakot

Several studies investigating potential earthquake frequency changes use declustered earthquake catalogs. However, declustering is an ill-posed problem that does not have a unique solution^52^ and that will lead to method-dependent results. Yet, to test the impact of potential earthquake clustering on our results, we have applied the traditional Reasenberg declustering algorithm^53^, obtained from the ZMAP toolbox^54^, to the CWB earthquake catalog. This deterministic algorithm aims to remove earthquake sequences, defined as chains of connected earthquakes in space and time, leaving only the initial earthquake in a given sequence. We use standard parameters^52^ adapted for Taiwan with ${\uptau }_{min}=1$ day the minimum look-ahead time for for building clusters when the first event is not clustered, ${\uptau }_{max}=10$ days, the maximum look-ahead time for building clusters, ${\text{p}}_{clust}=0.95$, a confidence probability, ${\text{x}}_{meff}=2.0$, the effective lower cutoff magnitude chosen here to be consistent with the CWB catalog, ${\text{x}}_{k}=0.5$, the increase in lower cutoff magnitude during clusters, and ${\text{r}}_{fact}=10$, the number of crack radii surrounding each earthquake within new events considered to be part of the cluster. We emphasize here that declustering has been performed over the entire catalog, without any regional selection of the seismicity (i.e. not only in the landsliding zone and not only above 15 km of depth), to prevent potential declustering biases associated to earthquake censoring.

Supplementary Figure S9 shows the influence of declustering on earthquake frequency, b-value, depth distribution and the probability of earthquake frequency change after Morakot in the landsliding zone (< 15 km depth). We observe that declustering mainly leads to a decrease of earthquake frequency over the 2.5 years before typhoon Morakot, and to very minor changes after. We find that declustering even slightly enhances the probability of an increase in earthquake frequency after Morakot. In addition, declustering does not significantly changes the time variation in b-value nor the depth-distribution of seismicity. We note that the Reasenberg declustering approach is well-suited to remove earthquake sequences that lead to significant changes (e.g. an aftershock sequence after a large mainshock) and not to identify potential triggered events during quiet periods associated with a constant and low frequency of earthquakes^48^. Under this potential limitation, this analysis demonstrates that earthquake clustering is not the reason for the observed changes after typhoon Morakot.

In addition, we are confident that large and deep mainshocks occurring in the landsliding zone, below 15 km, such as Mw 6.3 Jiashian earthquake in 2010, are not the cause for the observed seismicity changes. Indeed, declustering is efficient to remove most of the aftershocks caused by the Jiashian earthquake, which appear to be concentrated at depth between 15 and 30 km (Supplementary Fig. S10). To confirm this inference, we vary the values of the declustering parameters using a range of acceptable values around the reference values^48,55^: ${\uptau }_{min}=0.5{-}2.5$ days, ${\uptau }_{max}=3{-}15$ days, ${\text{p}}_{clust}=0.90{-}0.99$, ${\text{x}}_{k}=0{-}1$ and ${\text{r}}_{fact}=5{-}20$. We use a random sampling of the parameters space within these ranges, using a Monte-Carlo approach, to define 50 declustering parameter combinations. We obtain similar results, in terms of earthquake depth-distribution (Supplementary Fig. S9) and temporal variations of earthquake frequency (Supplementary Fig. S10) as in the reference declustering model. This confirms the robustness of the observed changes in shallow seismicity after Morakot that are not related to earthquake interactions.

### Seismicity temporal variation: random hypothesis versus significant regional seismicity change versus local earthquake interactions

Having a declustered catalog should theoretically imply that all the earthquakes are seismically independent from each other. In turn, earthquakes should be randomly distributed in time, unless a non-seismic process triggers them. Here we test this hypothesis by comparing the observed changes, in earthquake frequency and b-value after Morakot, obtained from the “true” declustered catalog with those from 200 “synthetic” earthquake catalogs over the period 2006–2015. Each synthetic catalog is generated using the exact same earthquakes as in the true declustered catalog, including their magnitudes, but the time of occurrence of each earthquake is randomly sampled over the period of interest (2006–2015) using a time step of 1 s. It results that each synthetic catalog has the exact same magnitude distribution and average earthquake frequency than the true one, but the temporal distribution of earthquakes is randomly distributed. For each catalog (true or synthetic), the probability of frequency change and the change in b-value after Morakot are computed by comparing the earthquakes occurring in the 2.5 years after and before Morakot (see Supplementary Fig. S12). The probability of a frequency change after Morakot for all the synthetic catalogs drops around a ratio 0.8–1.2, centered around 1, meaning there is no significant frequency change. This clearly departs from the frequency change observed using the true catalog that drops around a ratio of 1.3–1.6. In addition, the change in b-value of the true catalog is significantly greater than the changes of all the synthetic catalogs. Overall, these results mean that both the frequency change and the b-value increase after Morakot are robust features, that depart from random changes. In addition, because we performed these tests using the declustered catalog, which should only include independent earthquakes, this means that these robust and non-random changes should not be associated to the occurrence of large mainshocks. We also note that potential earthquake interactions in a local subset of the landsliding zone^56^ cannot explain the increase in earthquake frequency after Morakot (Supplementary Fig. S13).

## Supplementary information

Supplementary Figures

## Data Availability

The datasets used in this study are provided along with the manuscript. The earthquake catalogue is also accessible through the Taiwan Central Weather Bureau (https://gdms.cwb.gov.tw). The rainfall data is also accessible through Data Bank for Atmospheric Research at the Taiwan Typhoon and Floods Research Institute (https://www.narlabs.org.tw/en). The landslide dataset is also available by request to O.M., P.M. and N.H.

## Code availability

The Matlab code used to perform the analysis and the figures of this manuscript, as well as the data needed to process the figures of this paper, are provided here: https://zenodo.org/record/3778355. The Reasenberg declustering algorithm is accessible through the ZMAP set of tools (https://www.seismo.ethz.ch/en/research-and-teaching/products-software/software/ZMAP/ and https://github.com/CelsoReyes/zmap7).
