# Supplementary material for: Earthquake statistics changed by typhoon-driven erosion
Source: Sci Rep. 2020 Jul 2;10:10899. doi: 10.1038/s41598-020-67865-y (PMC7331670; doi:10.1038/s41598-020-67865-y)
Supplement: Supplementary file 1 — Supplementary Figures [file 41598_2020_67865_MOESM1_ESM.pdf]

Supplementary material for article

EARTHQUAKE STATISTICS CHANGED BY TYPHOON-DRIVEN EROSION

AUTHORS:
Philippe Steer[1], Louise Jeandet[1], Nadaya Cubas[2], Odin Marc[3,4,5], Patrick Meunier[3,6], Martine Simoes[7], Rodolphe Cattin[8], J. Bruce H. Shyu[9], Maxime Mouyen[10], Wen-Tzong Liang[11], Thomas Theunissen[12], Shou-Hao Chiang[13] and Niels Hovius[3,4].

[1]Univ Rennes, CNRS, Géosciences Rennes - UMR 6118, F-35000 Rennes, France.
[2]Sorbonne Université, CNRS-INSU, Institut des Sciences de la Terre Paris, ISTeP UMR 7193, F-75005 Paris, France
[3]Helmholtz Centre Potsdam, German Research Center for Geosciences (GFZ), 14473 Potsdam, Germany
[4]Institute of Earth and Environmental Sciences, University of Potsdam, 14476 Potsdam-Golm, Germany
[5]Géosciences Environnement Toulouse (GET), UMR 5563, CNRS/IRD/UPS, Observatoire Midi-Pyrénées (OMP), 14 Avenue Edouard Belin, 31400 Toulouse, France
[6]Laboratoire de Géologie, École Normale Supérieure de Paris, 75231 Paris CEDEX 5, France
[7]Université de Paris, Institut de physique du globe de Paris, CNRS, F-75005 Paris, France
[8]Géosciences Montpellier, Université Montpellier and CNRS UMR5243, 34090 Montpellier, France
[9]Department of Geosciences, National Taiwan University, Taipei, Taiwan
[10]Department of Space, Earth and Environment, Chalmers University of Technology, Onsala Space Observatory, Onsala, Sweden
[11]Institute of Earth Sciences, Academia Sinica, Taipei, Taiwan
[12]Department of Earth Science, University of Bergen, N-5007 Bergen, Norway
[13]Center for Space and Remote Sensing Research, National Central University, Taoyuan City 32001, Taiwan
*philippe.steer@univ-rennes1.fr

Supplementary material includes thirteen figures.

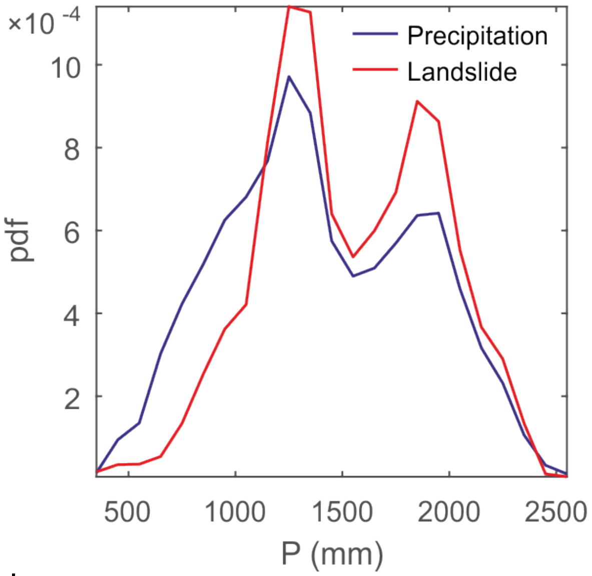

Supplementary Figure 1 | Influence of rainfall on landsliding during Morakot. Probability density functions (pdf) of cumulated rainfall *P* (blue line) in the landsliding zone and of cumulated rainfall at the location of the landslides (red line) during Morakot typhoon (7th to 9th of August 2009). Rainfall at the location of each landslide was obtained by natural-neighbor interpolation from local weather stations (see Methods).

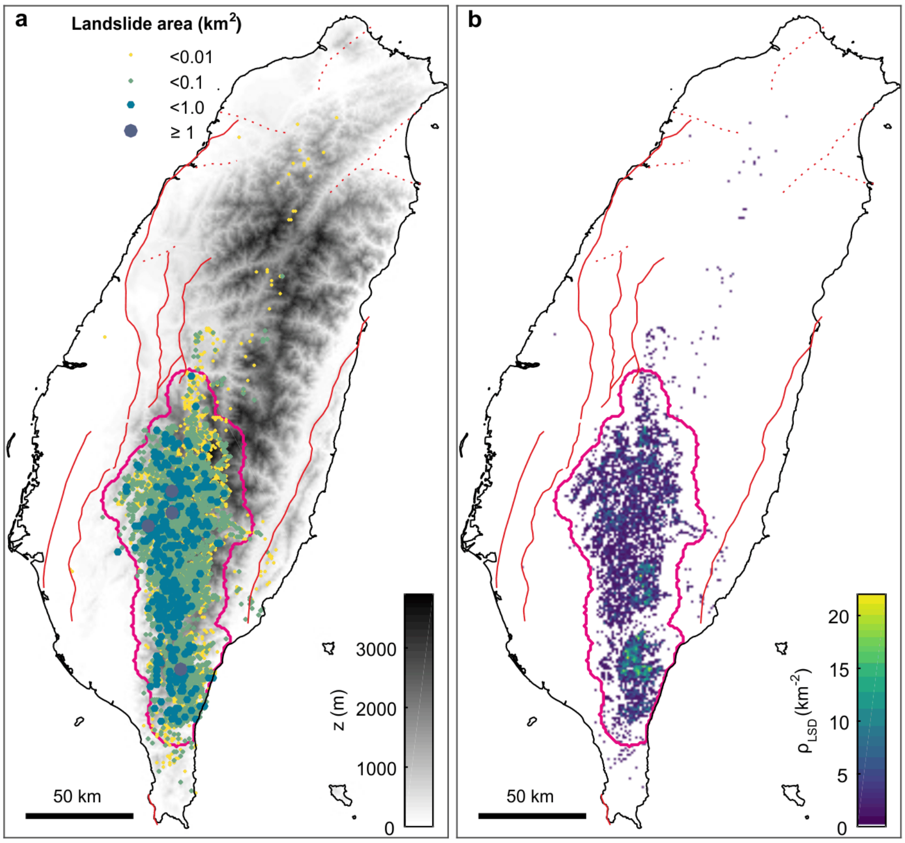

Supplementary Figure 2 | Morakot-driven landslides. a) Digital elevation model of Taiwan with location of the detected landslides triggered during Morakot typhoon. Circle size and color indicate the surface area of the landslides, while the magenta line bounds the area with a high spatial density of landslides (see Methods). **b**) Landslide spatial density estimated by dividing the number of landslides by the surface area of 1 km^2 pixels. The landsliding zone is bounded by the magenta contour line. Map were performed using Matlab R2019b.

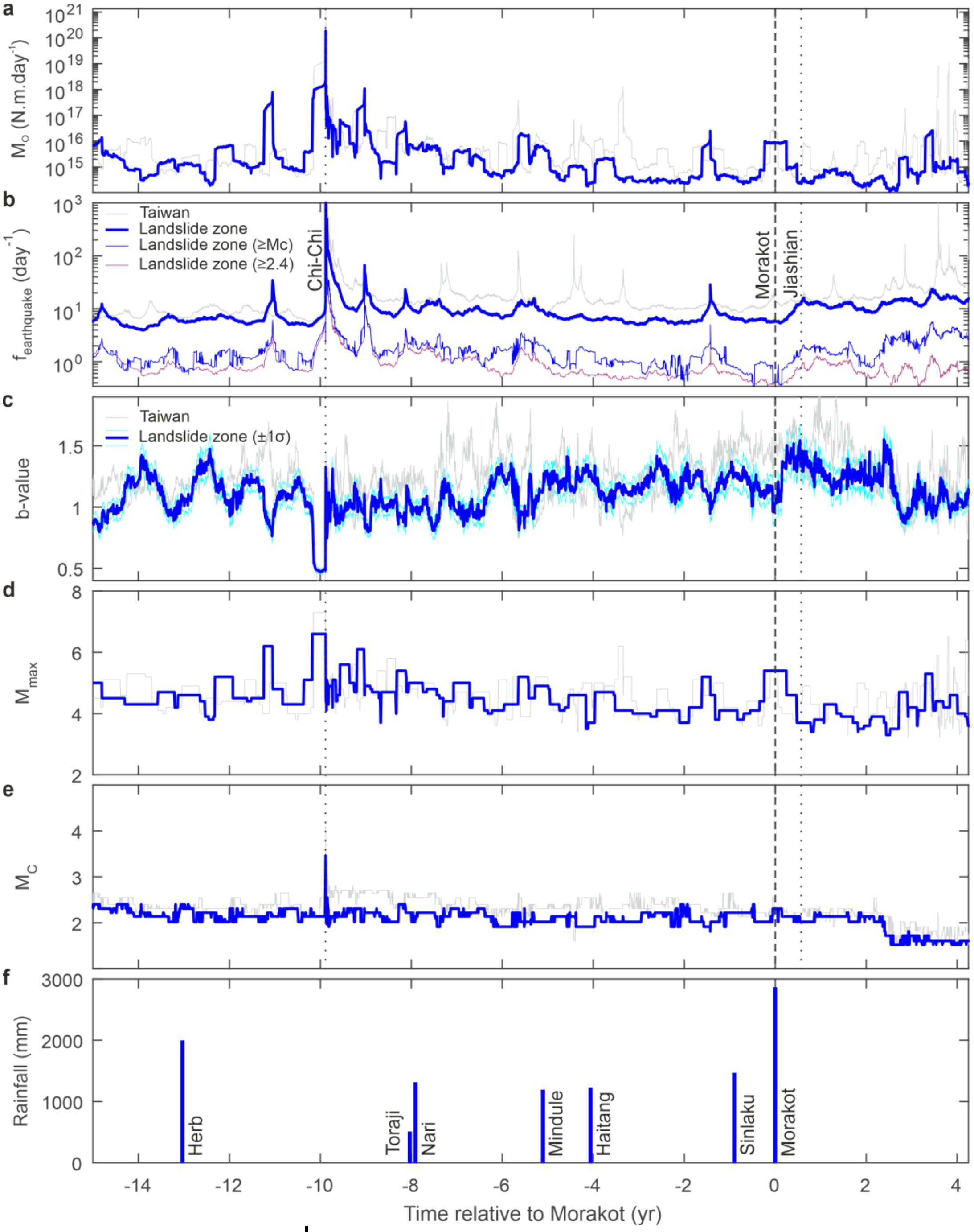

Supplementary Figure 3 │ Time evolution of seismicity in Taiwan relative to Morakot. Temporal evolution of shallow (<15 km) earthquake **a)** seimic moment rate, **b)** frequency, **c)** b-value of the Gutenberg-Richter law, **d**) maximum magnitude and **e**) completeness magnitude inside (heavy blue line) and outside (light grey line) the landsliding zone in time relative to Morakot. **f)** Cumulated rainfall during the major typhoons occurring over the same time period is indicated. The light blue line on panel a and b indicates the moment seismic rate or frequency of earthquakes greater than the completeness magnitude. All earthquake statistical parameters are computed using a sliding window considering N=1001 events.

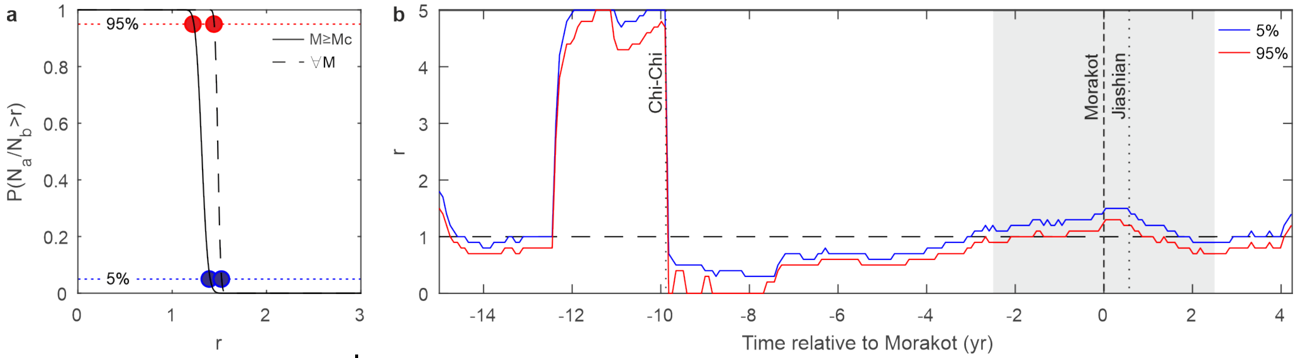

Supplementary Figure 4 | Probability of earthquake frequency change. a) Probability P of an earthquake frequency change of a ratio greater than r in the 2.5 yr after Morakot considering earthquakes of all magnitudes ($\forall M$, dashed black line) or only earthquakes above completeness magnitude ($\geq M_c$, solid black line). Red and blue dots indicate the bounds of the 90% confidence interval ($0.05 < P < 0.95$). **b**) Time variation of the 90% confidence interval on r. Except for Chi-Chi, Morakot (dashed vertical line) represents the only event associated with a significant and positive change of earthquake frequency over a period of 2.5 yr since 1994. The grey zone indicates the time when the analysis integrates events occurring simultaneously with Morakot.

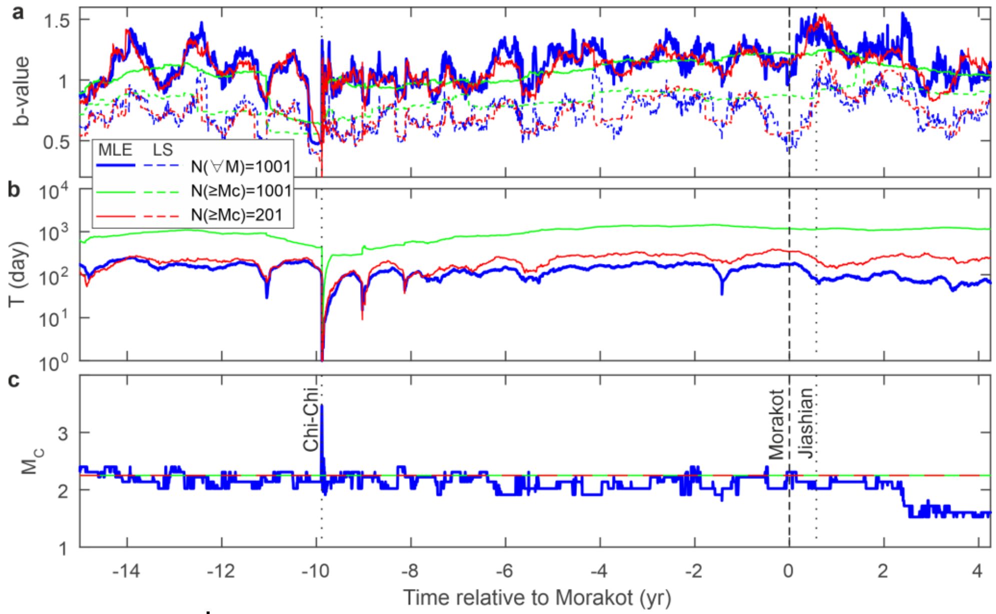

Supplementary Figure 5 | Sensitivity of b-value estimates to sampling and fitting methods. a) The temporal sliding window sample either a fixed number of earthquakes considering earthquakes of all magnitudes ($N(\forall M) = 1001$ events, blue line, same as the one in Fig. 2) or only earthquakes above completeness magnitude ($N(\geq M_c) = 1001$ or 201 events, green and red lines, respectively). **b**) Increasing the number of samples events increases the time duration T of the sliding window and smooths out temporal variations at high frequencies. **c**) In one method, the completeness magnitude M_c is determined from the sampled events (blue line) and varies with time, while in the other one the completeness magnitude is chosen *a priori*, here to 2.25 (red and green lines). On panel a) solid and dashed lines represent b-values obtained by maximum likelihood MLE or least-square LS fitting, respectively.

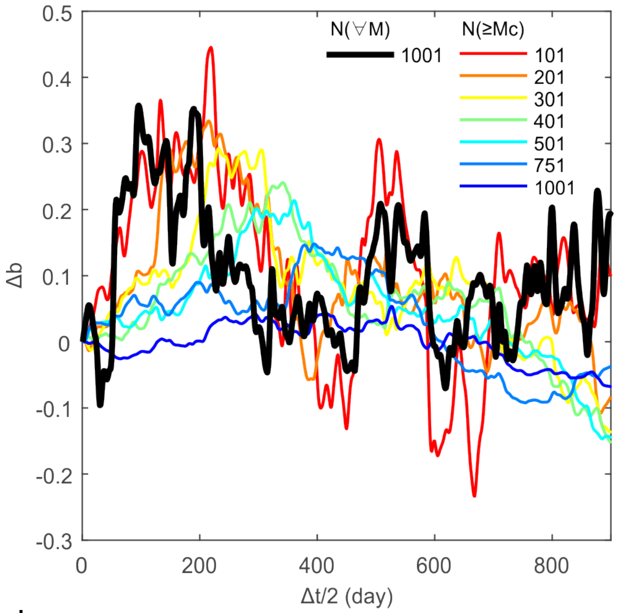

Supplementary Figure 6 | Sensitivity of b-value change after Morakot to sampling methods. Change in b-value estimate ($\Delta b = b(t_{Morakot} + \Delta t/2\,) - (t_{Morakot} - \Delta t/2\,)$) when comparing b-value at the date of Morakot $t_{Morakot}$ plus $\Delta t/2$ and minus $\Delta t/2$. The temporal sliding window samples either a fixed number of earthquakes considering earthquakes of all magnitudes ($N(\forall M) = 1000$ events, black bold line, same as the bold blue line in Fig. 2) or only earthquakes above completeness magnitude ($N(\geq M_c) = 101$ to 1001 events, red to blue lines, respectively). For the latter, M_c is chosen at 2.25.

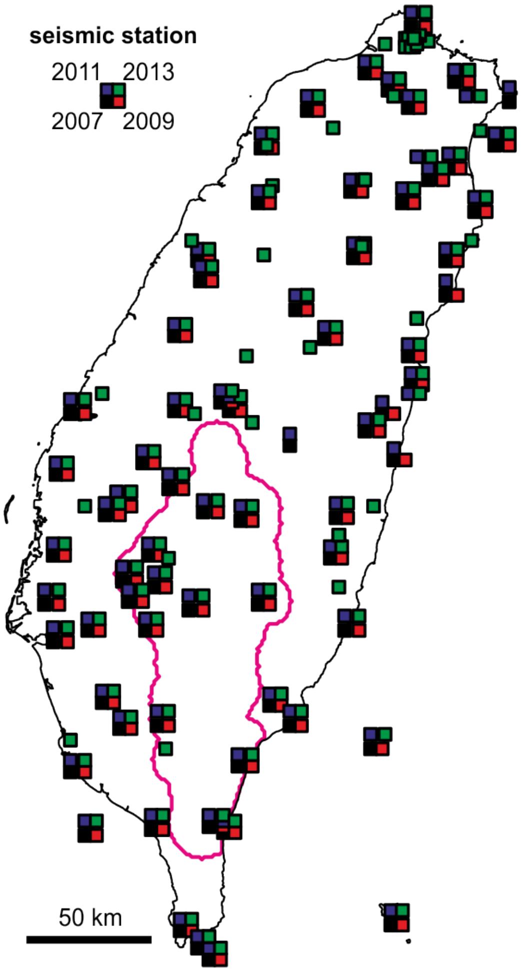

Supplementary Figure 7 │ Seismic network configuration and evolution. The location of seismic stations is shown by colored squares for 2007 (black), 2009 (red), 2011 (blue) and 2013 (green). Map was performed using Matlab R2019b.

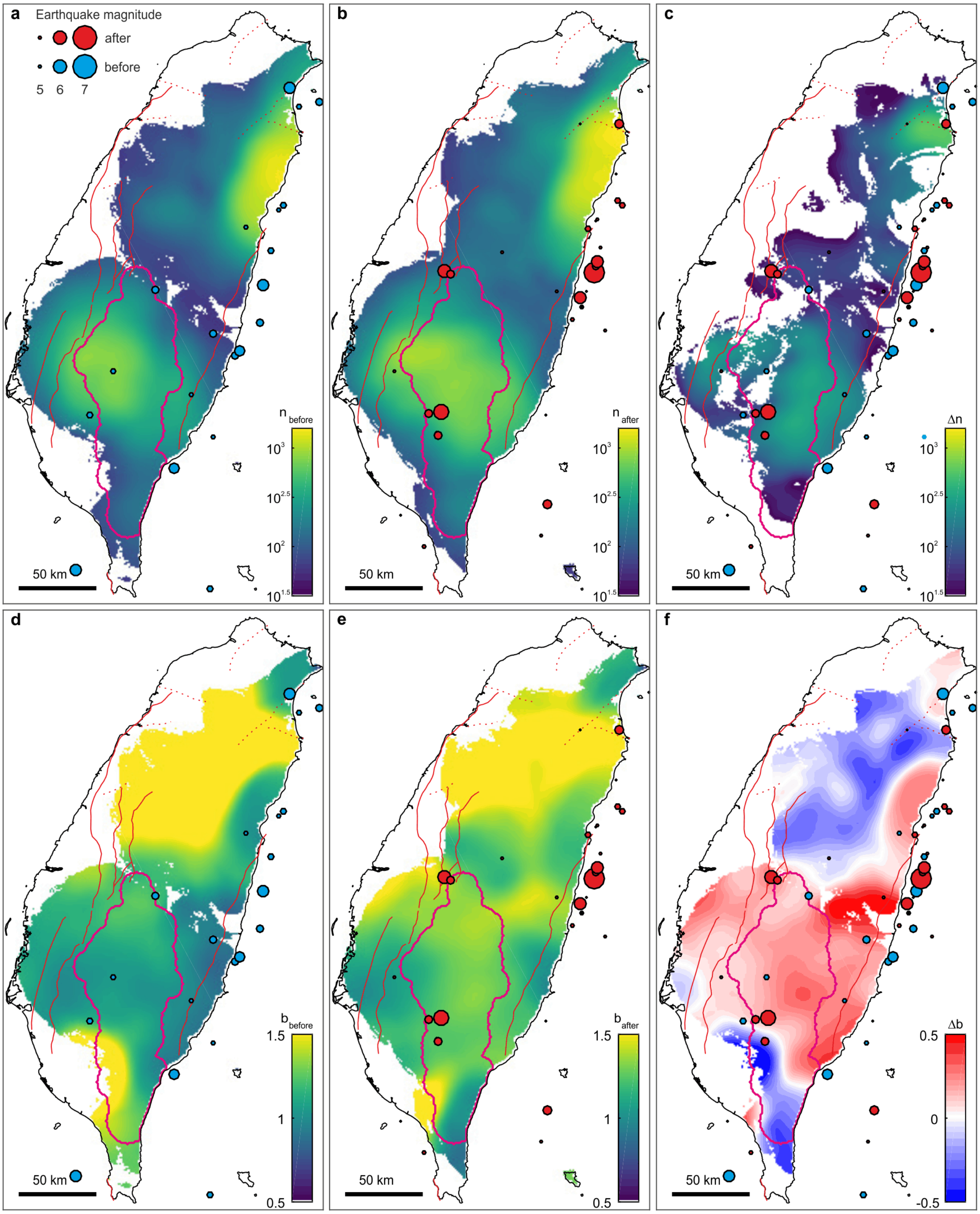

Supplementary Figure 8 | Changes in seismicity after Morakot. Maps of the of shallow (<15 km) earthquake number that have occurred during the 2.5 years **a**) before Morakot (n_{before}), **b**) after Morakot (n_{after}) and **c**) of the difference $\left(\Delta n = n_{after} - n_{before}\right)$. Only earthquakes greater than the completeness magnitude Mc were considered. **d-f**) Same than **a-c**) but for the b-value **d**) before Morakot (b_{before}), **e**) after Morakot (b_{after}) and **f**) the difference $\left(\Delta n = n_{after} - n_{before}\right)$. Maps were performed using Matlab R2019b.

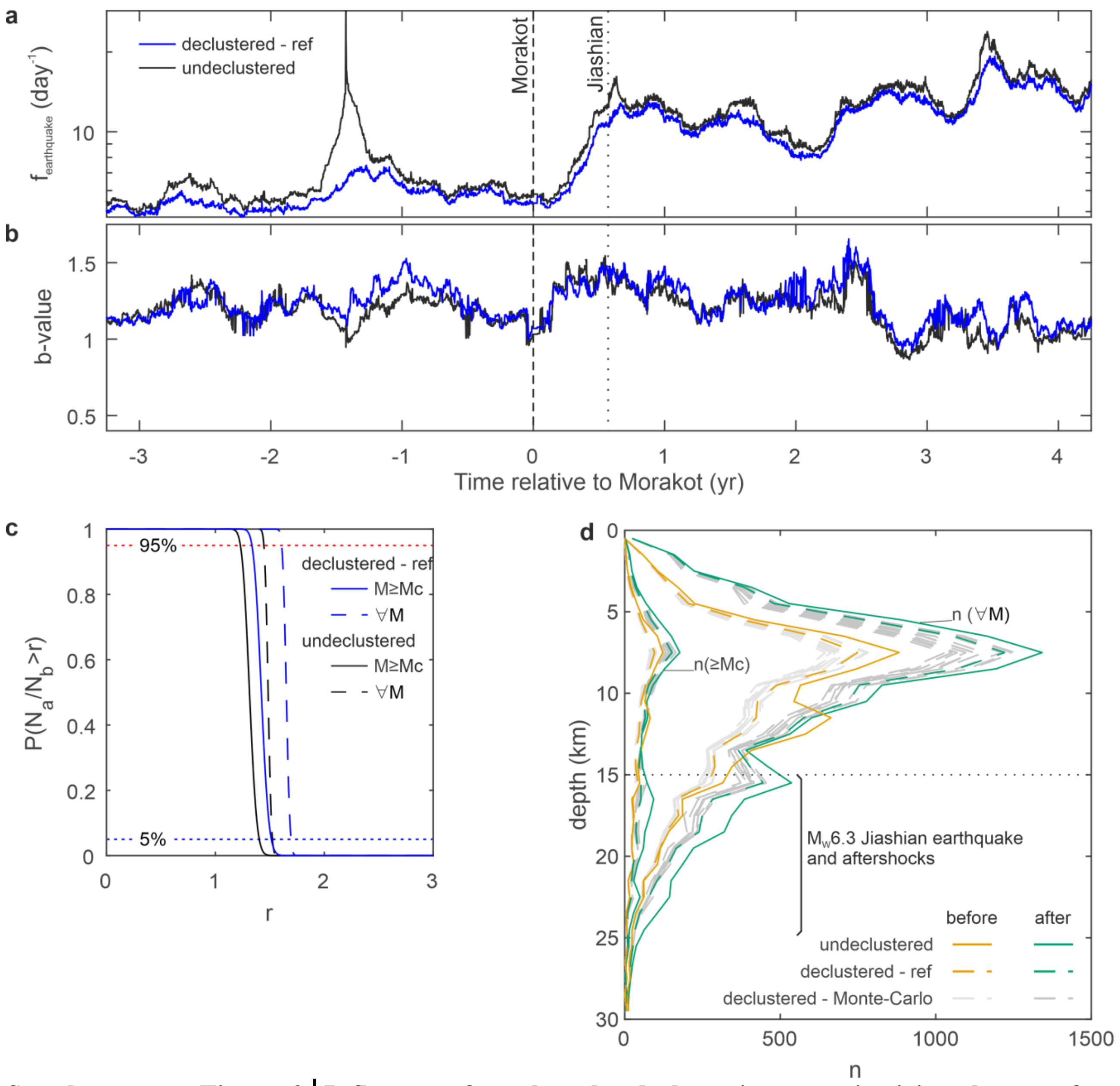

Supplementary Figure 9 | Influence of earthquake declustering on seismicity change after Morakot. Temporal evolution of shallow (<15 km) earthquake **a**) frequency and **b**) b-value of the Gutenberg-Richter law for the declustered (blue line) and undeclustered (black line) catalogs in the landsliding zone. **c**) Probability P of an earthquake frequency change of a ratio greater than r in the 2.5 yr after Morakot considering earthquakes of all magnitudes ($\forall M$, dashed black line) or only earthquakes above completeness magnitude ($\geq M_c$, solid black line). Same color convention for the declustered (blue line) and undeclustered (black line) catalogs. **d**) Histograms of event numbers versus earthquake depth during the 2.5 years before (yellow) and after (green) typhoon Morakot in the landsliding zone. Solid and dashed lines indicate the undeclustered and the reference declustered catalog, respectively, while the bold or light lines indicate the use of all magnitudes or only magnitudes greater than the completeness magnitude, respectively. Results obtained using the 50 declustered catalogues, resulting from a Monte-Carlo sampling of the model parameter space, are shown in light and dark grey.

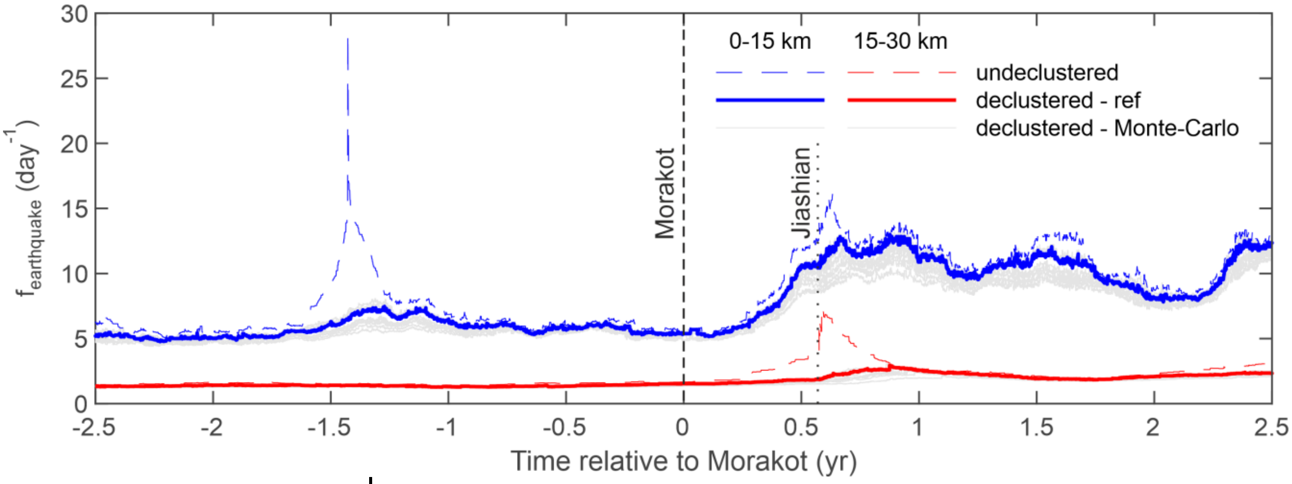

Supplementary Figure 10 | Impact of declustering on shallow and deep seismicity. Temporal evolution of shallow (<15 km; in blue) and deep (15-30 km; in red) earthquake frequency using the reference declustered (solid lines) or the undeclustered (dashed lines) catalogs in the 2.5 years after and before Morakot. Results obtained using the 50 declustered catalogues, obtained with a Monte-Carlo sampling of the model parameter space, are shown in light grey.

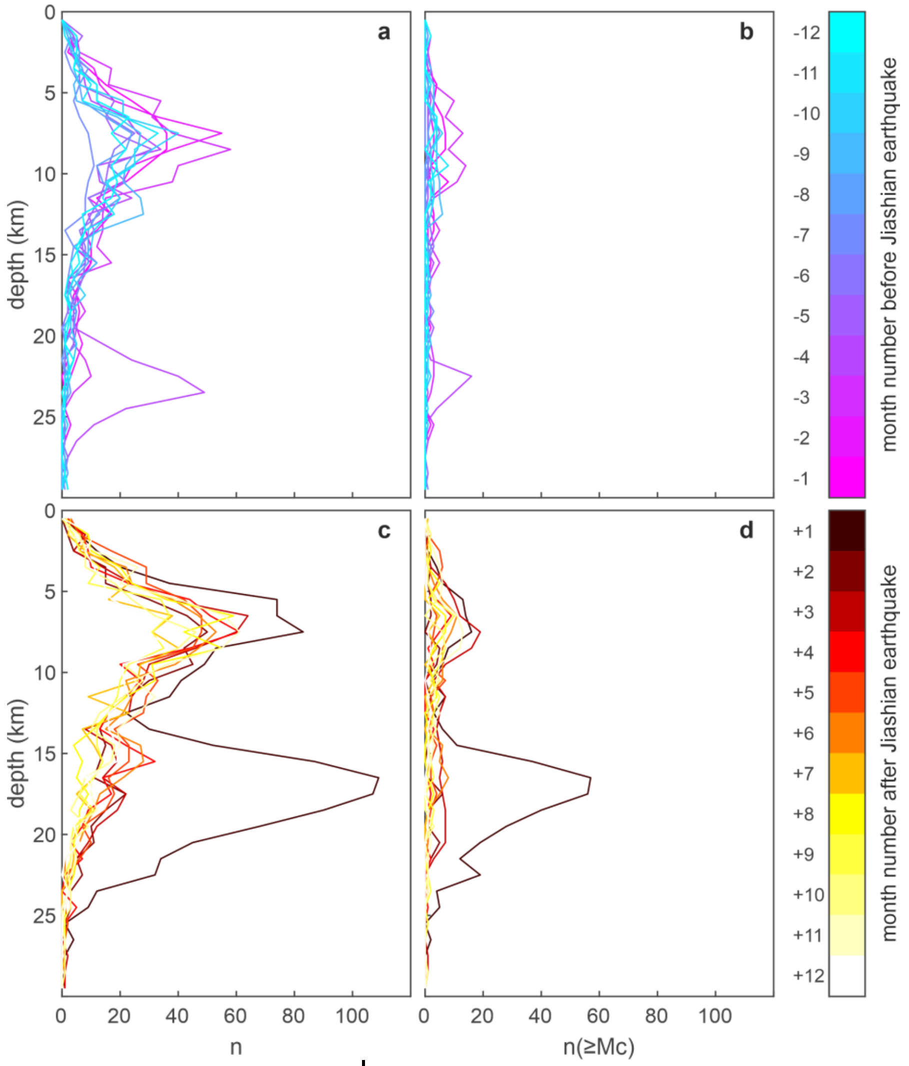

Supplementary Figure 11 | Impact of the Mw6.3 Jiashian earthquake on the depth distribution of seismicity. Monthly depth distributions of earthquakes inside the landsliding zone before **(a, b)** and after **(c, d)** Jiashian earthquake. We have considered either **(a, c)** all earthquakes or **(b, d)** only earthquakes with a magnitude greater than the completeness magnitude. The bin size is one kilometer along the depth axis. The line color indicates the month after or before Jiashian earthquake that occurred on the 4th of March 2010.

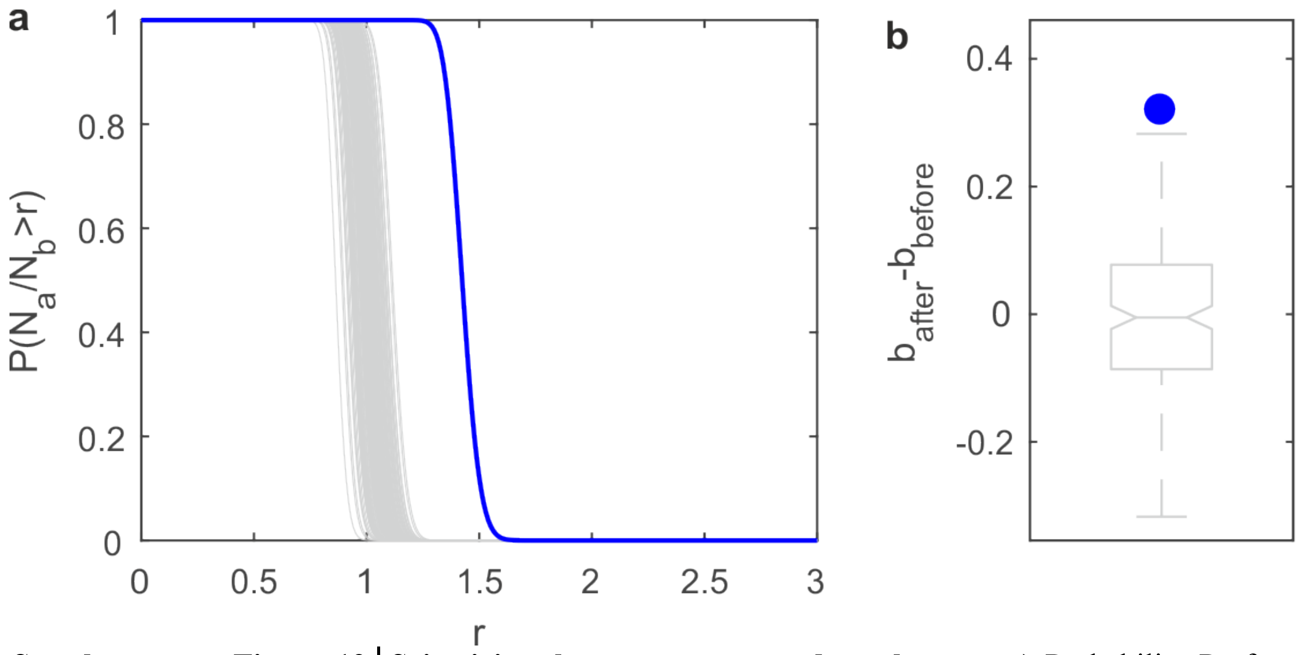

Supplementary Figure 12 | Seismicity changes versus random changes. a) Probability P of an earthquake frequency change of a ratio greater than r in the 2.5 yr after Morakot (compared to the 2.5 years before) using the true declustered catalog (blue line) or 200 synthetic ones (grey lines) that share the same average statistical properties. **b)** b-value change in the 2.5 yr after Morakot using the true declustered catalog (blue dot) or the synthetic ones, shown by a whisker plot.

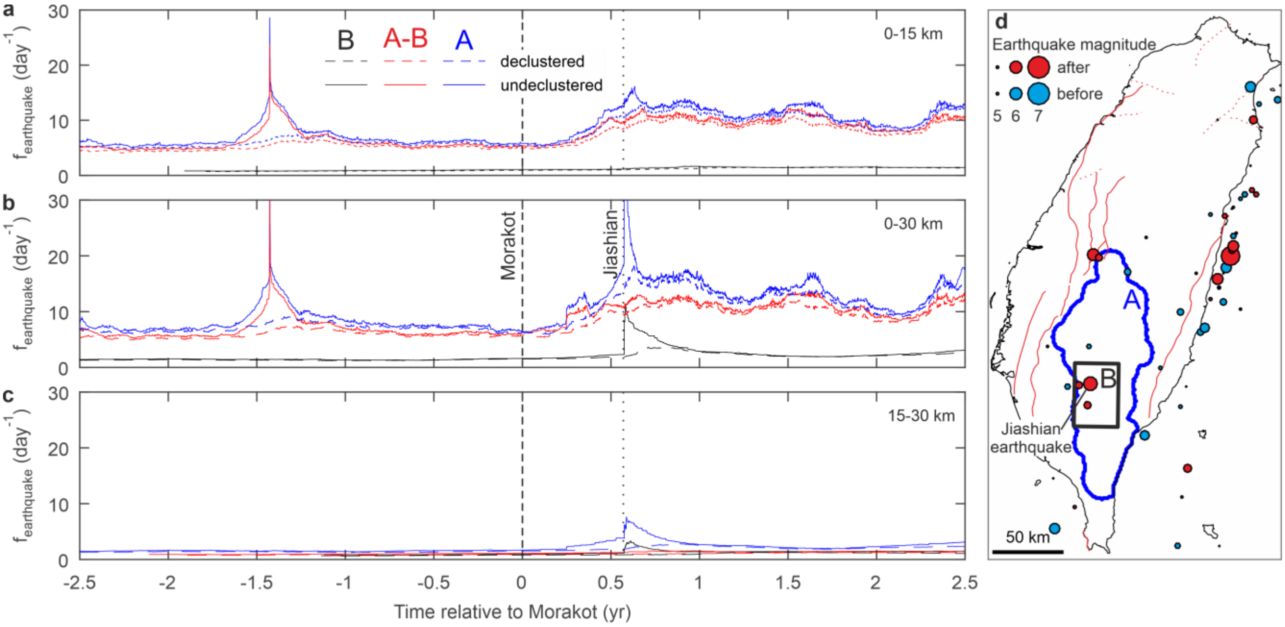

Supplementary Figure 13 | Earthquake interactions versus regional seismicity change. Temporal evolution of b) all, a) shallow and c) deep earthquake frequency in the landsliding zone (A, blue), in zone[56] (B, black) and in A minus B zone (A-B, red). Declustered (dashed lines) or undeclustered (solid lines) catalogs are indicated. d) Locations of the A and B zones. "After" and "before" refers to typhoon Morakot. Ref. 56 studied earthquakes statistics within zone B and suggested that the temporal variability of the seismicity in this area was due to the static stress changes from three M≥5.5 earthquakes: Jiashian (2010), Taoyuan (2010) and Wutai (2012). However, when excluding zone B from our statistics (bounded to zone A), our results remain unchanged. Therefore, the increase of seismicity and b-value changes that we evidenced in this study cannot be explained only by local earthquake interactions, which are restricted to zone B. Map on panel d was performed using Matlab R2019b.
